# Fasting Versus Post-Challenge Triglycerides and Pre-Existing Cavitating Lacunes: A Berlin “Cream & Sugar” Substudy

**DOI:** 10.3389/fneur.2013.00092

**Published:** 2013-07-10

**Authors:** Christopher O. Leonards, Li Wang, Jochen B. Fiebach, Matthias Endres, Martin Ebinger

**Affiliations:** ^1^Center for Stroke Research Berlin (CSB), Charité – Universitätsmedizin, Berlin, Germany; ^2^International Graduate Program Medical Neurosciences, Charité – Universitätsmedizin, Berlin, Germany; ^3^Klinik und Hochschulambulanz für Neurologie, Charité – Universitätsmedizin, Berlin, Germany; ^4^Excellence Cluster NeuroCure, Berlin, Germany; ^5^German Center for Cardiovascular Research (DZHK), Charité – Universitätsmedizin, Berlin, Germany

**Keywords:** ischemia, cerebral lacunes, leukoaraiosis, white matter disease, triglycerides, glucose

## Abstract

**Background and Purpose:** Although the presence of cavitating lacunes on brain imaging may have prognostic implications, the modifiable risk factors underlying these frequently observed lesions are not completely understood. We sought to determine if fasting and post-challenge triglycerides associate with cavitating lacunes.

**Methods:** All first ischemic stroke patients who completed a novel combined oral triglyceride and glucose tolerance test and MRI between January 2009 and June 2012 were included. Fluid-attenuated inversion recovery or T2 MRI sequences were used to visualize cavitating lacunes and white matter hyperintensities, which were graded using the Wahlund visual scale.

**Results:** One hundred and ninety patients were included (median age 66, IQR 52–73; 33% female; median National Institute of Health Stroke Scale 2, IQR 1–4). A forward stepwise binary logistical regression analysis applying the Hosmer–Lemeshow goodness of fit test adjusted for parameters significant in univariate analyses (at the *p* < 0.10 level) revealed that Wahlund scores (Wahlund 0–4: reference; Wahlund 5–10: adjusted odds ratio, 5.1; 95% confidence interval, 1.3–20.0, *p* = 0.019; Wahlund>10: adjusted odds ratio 9.6; 95% CI, 1.55–59.35; *p* = 0.015) and the highest quartile of post-challenge triglycerides (>295 mg/dL; adjusted odds ratio, 7.36; 95% confidence interval 1.24–43.70; *p* = 0.028) independently associated with the presence of cavitating lacunes.

**Conclusion:** Post-challenge serum triglycerides are independently associated with the presence of cavitating lacunes.

## Introduction

Although cavitating lacunes (small, deep 3–20 mm cerebrospinal fluid containing “holes”) (1) often appear “silently” (without stroke-like symptoms) on brain imaging (2), their presence is thought to more than double the risk of stroke and dementia (3). Age and hypertension are two established risk factors for cavitating lacunes (2–4), but evidence suggests that elevated serum triglycerides may also be a key factor in cavitating lacune development and progression (2).

Postmortem findings support the hypothesis that triglycerides play a role in cavitating lacunes. First, histopathology studies have identified fat-laden macrophages surrounding the small vessels in areas of cavitating lacunes (5) similar to that of the lipid-laden monocytes observed in the “fatty streak” of muscular arteries affected by atherosclerosis (6). It has been proposed that prolonged exposure to elevated triglycerides alters endothelial metabolism and expression of binding molecules thereby predisposing for monocyte aggregation and forming the nidus of a “fatty streak” (7). Second, studies have determined that vessels supplying cavitating lacunes are frequently affected by “lipohyalinosis” (fatty hyaline build-up within the vessel wall), a specific subgroup of the broader “small vessel disease” term (8).

Elevated triglycerides have long been suspected of being a modifiable cerebrovascular risk factor (9–11), however findings from studies that have measured fasting triglycerides have yielded conflicting results (12). Traditionally measured in the fasting state to avoid variability, more recent findings from large epidemiological studies (with over 10,000 participants) indicate that post-fat challenge triglyceride levels may be a more reliable indicator of cardiovascular and in particular ischemic stroke risk than fasting levels (10, 11). This has led to the recommendation of possibly introducing a standardized oral triglyceride tolerance test to clinical practice (13).

We therefore sought to determine if an association exists between fasting and post-challenge triglyceride levels and cavitating lacunes.

## Materials and Methods

### Participants

The Berlin “Cream & Sugar” Study (NCT 01378468) is an ongoing prospective cohort study that aims to assess the prognostic impact of post-challenge triglyceride and glucose levels in ischemic stroke patients. Detailed methods and inclusion/exclusion criteria are presented elsewhere (14). Briefly, we screened all male and female first-time acute ischemic stroke patients (3–7 days after symptom onset) over 18 years of age, who were admitted to one of three university campus hospitals in Berlin. Ischemic stroke was defined as a focal neurological deficit lasting for at least 24 h with no signs of hemorrhage on cerebral imaging. First ever ischemic stroke was defined here using relevant medical history and disregarded potential “silent” strokes on cerebral imaging that may have occurred prior to study enrollment. All suspected ischemic strokes were verified radiologically using diffusion-weighted images (DWI). Stoke was categorized according to a mechanism-based classification scheme (Trial of ORG 10172 in Acute Stroke Treatment, or TOAST) (15). All patients provided informed consent and the study was approved by the local ethics committee.

Stroke severity was assessed 3–7 days after first ischemic stroke using the National Institute of Health Stroke Scale (NIHSS).

### Oral triglyceride tolerance test

Fasting blood samples were drawn at 8 a.m. Directly thereafter, patients drank 250 mL of 32% fat cream within 30 min in the presence of a Center for Stroke Research Berlin staff member to ensure that the cream was ingested. Three hours later (11 a.m.), a second blood draw was performed and was immediately followed by a standard 75 g oral glucose tolerance test. Subsequent blood draws were then performed at 12 p.m. and 1 p.m.

### Blood samples

Baseline data and venous blood samples were collected after participants had provided informed consent and fasted overnight for ≥12 h. Fasting blood samples were drawn at 8 a.m. Triglyceride and cholesterol concentrations were determined in freshly drawn venous blood samples enzymatically using a Cobas 6000 analyzer (Roche/Hitachi). Glomerular filtration rate (GFR) was estimated using the Modification of Diet in Renal Disease (MDRD) (16) formula according to relationship 186 × serum creatinine^−1.154^ × age^−0.203^ × 1.210 (if Black) × 0.742 (if female). Diabetes was defined as current use of antidiabetic medication, serum glycosylated hemoglobin (HbA1C) of>6.5% or 2 h oral glucose tolerance test value of>200 mg/dL. Hypertension was defined as current antihypertensive medication use. Hyperlipidemia was defined as fasting total cholesterol ≥200 mg/dL and/or fasting triglycerides ≥150 mg/dL and/or use of lipid lowering medications prior to ischemic stroke (17).

### Image acquisition, cavitating lacunes, and WMH severity

MRI was performed using both 3 T (Tim Trio; Siemens AG, Erlangen, Germany) and 1.5 T (Avanto, Siemens Medical Solutions, Erlangen, Germany) scanners. Patients who were admitted to one of three university campus hospitals underwent “Fast Track” Stroke MRI that consisted of DWI, T1, T2^∗^, and either FLAIR or T2 weighted images (depending on the campus hospital). FLAIR or T2 weighted images were used to assess cavitating lacunes and white matter hyperintensity (WMH) severity. Because different campus hospitals utilized different MRI scanners (3 and 1.5 T), sensitivity of MRI field strength to detect lacunes and WMH was tested using Fischer’s exact (lacune presence) and Mann–Whitney *U* tests (non-stratified Wahlund scores).

**Figure 1:**
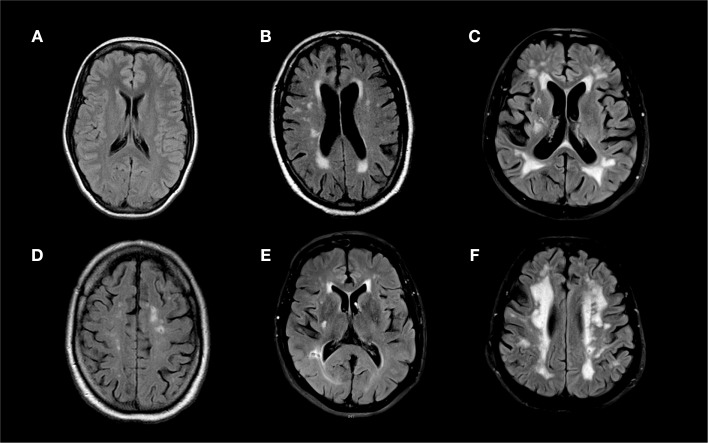
**Fluid attenuated inversion recovery (FLAIR) MRI sequences of (A) a 21 year old patient with an overall Wahlund score of 0, (B) a 51 year old patient with an overall Wahlund score of 8, (C) a 69 year old patient with a Wahlund score of 20, (D) a 75 year old man with an overall Wahlund score of 12 and a 4 mm cavitating lacune in the left parietal lobe, (E) a 63 year old man with an overall Wahlund score of 5 and a 5.5 mm cavitating lacune in the right occipito-temporal region, (F) a 70 year old man with an overall Wahlund score of 20 and an 4 mm cavitating lacune in the left pareito-temporal region**.

White matter hyperintensity were scored according to the Age-Related White Matter Change (18) and Fazekas (19) visual classification systems (Figure 1). In the Wahlund classification system, FLAIR and T2 image hyperintensities are rated from 0 to 3 based on size and confluence of the lesions on both the right and left sides of the brain in the following pre-specified regions: frontal, parieto-occipital, temporal, infratentorial/cerebellum, and basal ganglia. The final score is the sum of all regions and ranges from 0 (no WMH) to 30 (most severe WMH). In the Fazekas classification system, the image with the most severe WMH is rated on a scale of 0 to 3 [0, no WMH; 1, punctate foci; 2, beginnings of confluent foci; 3 large confluent areas; (19)].

Cavitating lacunes were defined as small (3–20 mm in all directions) hypointense lesions on FLAIR and hyperintense lesions on T2 weighted images with a corresponding low intensity area on T1 weighted images (Figure 1) (1, 20). Cavitating lacunes were identified as ovoid/spheroid shaped areas with similar signal intensity to that of cerebrospinal fluid generally (on FLAIR images) surrounded by a hyperintense rim (2). Because Virchow–Robin spaces also have similar signal intensity to cerebrospinal fluid, we differentiated cavitating lacunes from Virchow–Robin spaces based on size [cavitating lacunes: 3–20 mm; Virchow–Robin spaces smaller than 3 mm and generally following a vessel; (2)]. We recorded both presence of cavitating lacunes (present versus not present) and the number of cavitating lacunes observed in each patient.

### Statistical analysis

Continuous variables were tested for normal distribution using the Kolmogorov–Smirnov test. Mann–Whitney *U* tests were used to examine relationships between baseline and serum parameters (not normally distributed) and lacunes (present or not present). The relationship between categorical variables (gender, hypertension, smoking history, and diabetes mellitus), lacunes, and WMH severity was assessed using two-sided Fisher’s exact tests. If a significant relationship was found, *post hoc* analyses using Sidak’s method were performed (level of significance at 0.017). To estimate odds ratios in the categorical analysis, Wahlund scores were stratified into 3 groups: mild (Wahlund score of 0–4), moderate (Wahlund: 5–10), and severe (Wahlund:>10), as this classification (21, 22) and very similar stratification schemes have been used previously (23, 24) and seem to correspond well with standard visual classification systems (23). Spearman’s correlation coefficients were calculated to assess relationships between number of lacunes and baseline/serum parameters.

We performed a forward stepwise binary logistical regression analysis using the Hosmer–Lemeshow goodness of fit test. This analysis included all parameters associated with cavitating lacunes (at the *p* < 0.10 level) from univariate analyses and the dichotomized categorical variable cavitating lacunes (present versus not present). Serum glucose, glycosylated hemoglobin, creatinine, C-reactive protein, waist circumference, waist-to-hip ratio, and systolic blood pressure were not normally distributed and were stratified into quartiles. Age was categorized by decades starting at age 18 years and ending at age 101 years to incorporate all patients enrolled in our study. All statistical analyses were performed using SPSS 19.

## Results

We enrolled 211 patients in the Berlin “Cream & Sugar” study between January 2009 and June 2012. Of those, 190 patients (median age 66, IQR 52–73; 33% female; median NIHSS 2, IQR 1–4) had both MRI (148 FLAIR, 42 T2) and serum parameters available. All strokes were confirmed on MRI.

Results from univariate analyses and patient demographics for cavitating lacunes are presented in Table 1. Patients who had more cavitating lacunes were older (Spearman’s rho = 0.118, *p* = 0.042), more likely to have higher post-challenge glucose levels (Spearman’s rho = 0.121, *p* = 0.050), and had higher Wahlund (Spearman’s rho = 0.305, *p* < 0.01) and Fazekas scores (Fischer’s exact, *p* < 0.01). *Post hoc* testing revealed that patients with Fazekas scores of 2 and 3 had significantly more lacunes than patients with Fazekas scores of 1 (Fazekas 2: *p* = 0.031, 95% confidence interval 0.15–0.40; Fazekas 3: *p* < 0.01, 95% confidence interval 0.15–0.68). Although the associations did not reach significance, patients with more cavitating lacunes tended to have higher glycosylated hemoglobin (HbA1c, Spearman’s rho = 0.117, *p* = 0.054). In patients with HbA1c values<6.5%, elevated absolute 5 h post-challenge triglycerides significantly associated with lacune presence (*p* = 0.006) and number of lacunes (*p* = 0.019). Moreover, in patients with HbA1c values<6.5%, higher triglyceride area under the curve significantly associated with lacune presence (*p* = 0.032). Number of lacunes, however, was not significantly associated with triglyceride area under the curve in these patients (*p* = 0.134).

**Table 1:** **Demographic characteristics according to presence of cavitating lacunes**.

	No lacunes	Lacunes	Total	*p* Value
No. of participants	156	34	190	
Female, *n* (%)*	55 (30)	6 (3)	61 (33)	0.065
Age, year^†^	63 (51–72)	70 (61–75)	66 (52–73)	0.035
Hypertension, *n* (%)*	76 (48)	20 (13)	96 (61)	0.286
Hyperlipidemia, *n* (%)*	83 (48)	25 (15)	108 (63)	0.109
Current smokers, *n* (%)*	29 (19)	7 (5)	36 (23)	0.274
Diabetes, *n* (%)*	20 (13)	6 (4)	26 (17)	0.411
Wahlund score^†^	4 (1–5)	8 (3–11)	3 (1–6)	<0.01
NIHSS^†^	1 (0–2)	2 (0–3)	1 (0–2)	0.269
eGFR^†^	83 (72–100)	76 (69–96)	82 (71–98)	0.416
Creatinine, μmol/L^†^	79 (67–89)	85 (65–98)	80 (67–90)	0.272
Systolic BP^†^	130 (120–150)	140 (120–150)	135 (120–150)	0.601
Diastolic BP^†^	80 (70–87)	80 (70–85)	80 (70–86)	0.739
BMI, kg/m^2^^†^	26 (23–29)	26 (25–29)	26 (24–29)	0.759
LDL (mg/dL)^†^	110 (91–137)	112 (81–136)	110 (90–137)	0.558
CRP (mg/dL)^†^	0.3 (0.1–0.7)	0.2 (0.1–0.8)	0.3 (0.1–0.7)	0.696
HbA1c, %^†^	5.6 (5.2–6.1)	5.8 (5.5–6.2)	5.6 (5.3–6.1)	0.033
Waist-to-hip ratio^†^	0.96 (0.92–1.0)	0.98 (0.94–1.03)	0.97 (0.92–1.0)	0.092
Fasting Glc (mmol/L)^†^	5.3 (4.8–6.0)	5.7 (4.7–6.7)	5.3 (4.8–6.1)	0.220
3 h Glc (mmol/L)^†^	5.1 (4.8–5.7)	5.4 (4.9–6.6)	5.2 (4.8–5.8)	0.052
4 h Glc (mmol/L)^†^	7.2 (5.6–9.2)	7.8 (6.0–10.4)	7.3 (5.7–9.4)	0.271
Fasting TG (mmol/L)^†^	1.2 (0.9–1.6)	1.4 (1.1–1.7)	1.2 (1.0–1.6)	0.098
5 h TG (mmol/L)^†^	2.3 (1.7–3.1)	3.0 (1.8–3.9)	2.4 (1.7–3.4)	0.080

*Values are median (interquartile range) unless otherwise specified*.

*NIHSS indicates National Institute of Health Stroke Scale; mRS, modified Rankin Scale; eGFR, estimated glomerular filtration rate; BP, blood pressure; LDL, low density lipoprotein; CRP, C-reactive protein; HbA1c, glycosylated hemoglobin; Glc, glucose; TG, triglycerides*.

*Fischer’s exact test followed by Sidak’s post hoc method (if significant).

*^†^Mann–Whitney U test*.

Presence and number of lacunes and WMH did not significantly differ in patients rated with FLAIR versus T2 images (Fischer’s exact test: lacune presence, *p* = 0.66; number of lacunes, *p* = 0.68; WMH, *p* = 0.39). Comparisons across patients according to MRI strength (3 versus 1.5 T) revealed that those patients who received 3 T MRI scans (*N* = 95) were rated as having significantly more WMH than those scanned with a 1.5 T MRI (*N* = 94; *p* < 0.01). Dichotomized presence (present versus not present) and absolute number of cavitating lacunes did not differ according to MRI field strength (Exact Mann–Whitney *U*, cavitating lacune presence, *p* = 0.89; Exact Mann–Whitney *U*, number of cavitating lacunes, *p* = 0.589).

A forward stepwise binary logistical regression using the Hosmer–Lemeshow goodness of fit test that was adjusted for age (decades), NIHSS scores, fasting and post-challenge triglycerides and glucose, glycosylated hemoglobin, waist-to-hip ratio, and WML (Wahlund and Fazekas scores) revealed that WML (both Wahlund and Fazekas) scores and the highest quartile of post-challenge triglycerides independently associated with presence of cavitating lacunes (Table 2).

**Table 2:** **Results from forward stepwise binary logistical regression analysis assessing risk factors for cavitating lacunes**.

Predictors	OR	95% CI	*p* Value
Wahlund score 0–4			Reference
Wahlund score 5–10	5.101	1.30–20.02	0.019
Wahlund score>10	9.600	1.55–59.36	0.015
Fazekas score 0–1			Reference
Fazekas score 2	4.777	1.26–18.09	0.021
Fazekas score 3	6.932	1.06–45.45	0.044
TG<1.73 mmol/L			Reference
TG 1.73–2.36 mmol/L	0.547	0.07–4.59	0.578
TG 2.36–3.33 mmol/L	1.736	0.24–12.61	0.586
TG>3.33 mmol/L	7.358	1.24–43.70	0.028

*Odds ratios are adjusted for age (decades), NIHSS scores, fasting and post-challenge triglycerides and glucose, glycosylated hemoglobin, waist-to-hip ratio, and Wahlund and Fazekas scores.CI indicates confidence interval; OR, odds ratio; ref, reference; TG, triglycerides*.

## Discussion

The primary findings of this study were that elevated post-challenge serum triglycerides (>3.33 mmol/L) and WMH severity independently associated with cavitating lacunes. To our knowledge, this is the first study that has assessed the relationship between fasting and post-challenge serum triglycerides and pre-existing cavitating lacunes in acute ischemic stroke patients. Previous studies have found a relationship between non-fasting triglycerides and risk of ischemic stroke and heart disease (10, 11, 25). Our findings are similar in that post-challenge triglycerides independently associated with the presence of cavitating lacunes but fasting triglycerides did not.

Triglyceride concentrations rose markedly in all patients following the fat challenge. Interestingly, all patients showed very similar increases in triglyceride concentrations following the challenge until the 5 h post-challenge time-point, an occurrence that others have also observed (26). Groot et al. (26) measured triglyceride concentrations for 24 h following an oral fat challenge and noted that elevated triglycerides measured at the 8 h time-point or later were indicative of increased common carotid artery intima-media thickness. Though not statistically significant in their study (26), graphical representation showed that triglyceride levels began to diverge between those patients with and without carotid artery atherosclerosis at the 5 h time-point. Though our study was a relatively small retrospective sub-analysis, these findings could suggest that time to triglyceride “clearance” may play a role in cerebrovascular risk and that blood draws between 5 and 8 h post-challenge may be optimal for triglyceride analysis. Additionally, results from our univariate analysis indicate that a triglyceride tolerance test may be of particular benefit for the assessment of vascular risk in patients without diabetes mellitus. Further testing is needed to verify these finding.

Pathophysiologically, “silent” cavitating lacunes are thought to arise from deep cortical ischemia (5, 27, 28). A recent literature review concluded that the risk of ischemic stroke onset nearly doubles if cavitating lacunes are present on MRI (3). We found that post-challenge triglycerides associated with cavitating lacunes and others have identified a strong independent association between non-fasting triglycerides and ischemic stroke risk (10, 11). Taken together, it is possible that cavitating lacunes mark high stroke risk patients who may be identifiable in a cost-effective manner by measuring non-fasting triglycerides and even possibly benefited by triglyceride lowering strategies (life style modification or medication).

Our results conflict with those of Gouw et al. (2), who assessed possible risk factors for lacune progression (in 396 patients) and found that elevated fasting triglycerides and body mass index (BMI) were associated with the appearance of new cavitating lacunes. We found that obesity indices (BMI and waist-to-hip ratio) associated with WMH and both fasting and non-fasting triglycerides (data not shown) but, unlike Gouw et al. obesity indices did not correspond with the presence of cavitating lacunes. We expected obesity indices to associate with cavitating lacunes, as cavitating lacunes associated with non-fasting triglycerides and WMH. However, it is likely that our study was underpowered to detect these relationships, as there were only 34 participants with cavitating lacunes.

We focused on cavitating lacunes (as opposed to non-cavitating lacunes or a combination of the two) for the sake of simplicity as there is currently much debate regarding terminology. Because it remains unclear which lacunes will cavitate and which will not (or perhaps all cavitate after a certain time), the recommendation has been put forth to independently examine cavitating lacunes and WMH (which might be an early form of lacune that has not yet cavitated or something else altogether) and their respective risk factors (29). Our results show that WMH severity independently associates with the presence of cavitating lacunes, thus lending credence to the argument that though the exact etiologies of WMH and cavitating lacunes may differ, it is difficult to disaggregate the two (29). Moreover, this association may support the hypothesis that cavitating lacunes are simply a more pronounced form of WMH (29).

A distinction has been made between: (1) single, larger, symptomatic lacunar infarcts of microatheromatous origin and (2) small, multiple, typically asymptomatic lacunar infarcts (generally accompanying WMH) possibly of arteriolosclerosis origin (30). None of our patients reported “stroke-like symptoms” before the event leading to hospital admission and cerebral imaging was performed in the acute stroke setting. Because cavitation seems to occur slowly – taking well over a month post-insult for complete cavitation to occur in most cases (31) – and WMH associated with presence of lacunes so strongly, we believe the cavitating lacunes present in our patient sample likely arose from arteriolosclerotic (small vessel disease) origin rather than microatheromatous origin.

Our study has limitations. First, because this was a Berlin “Cream & Sugar” substudy, there was a selection bias toward mildly affected stroke patients (median NIHSS at hospital admission 2, range 0–24; median NIHSS at time of fat challenge 1, range 0–14). This resulted from the Berlin “Cream & Sugar” study’s relatively stringent inclusion criteria, as only those patients who: (1) were deemed capable of completing and tolerating a novel combined oral triglyceride/glucose tolerance test (i.e., patients without swallowing disorders, lactose intolerance, malabsorption, etc.) and (2) who did not have pre-existing conditions that could potentially influence lipid metabolism (liver disease, alcoholism, pancreatic disease, etc.) were selected. Second, the cohort was comprised primarily of older patients (median 66 years), which has been well established to associate with cavitating lacunes (3) and increased WMH severity (18). Third, women were underrepresented (33%). We therefore recommend a cautious interpretation of the observed trend across gender (Table 1) and lacune presence. Fourth, because we pooled patients that were admitted to various hospitals and scanned with different MRI field strengths (3 and 1.5 T), it is possible that lacunes and WMH were underreported. However, results from the logistical regression analysis did not change after we retrospectively controlled for MRI field strength. Finally, our cohort was relatively small (190 patients). We decided to use a forward stepwise algorithm for data analysis to minimize possible complications arising from low covariate incidence rates. However, larger studies are needed to assess the validity of our findings.

In conclusion, we assessed fasting and post-challenge triglyceride levels in acute ischemic stroke patients with and without pre-existing cavitating lacunes and found that elevated post-challenge triglycerides were associated with the presence of cavitating lacunes. Patients with elevated non-fasting triglycerides may be at a higher risk for cerebral ischemia and therefore benefit from triglyceride lowering strategies (life style modification or medication).

## Conflict of Interest Statement

The authors declare that the research was conducted in the absence of any commercial or financial relationships that could be construed as a potential conflict of interest.
